# Simulation-based education improves postpartum hemorrhage management knowledge and skills: a randomized controlled trial

**DOI:** 10.3389/fmed.2026.1939595

**Published:** 2026-09-29

**Authors:** Ömercan Aksoy, Ergül Aslan

**Affiliations:** 1 Faculty of Health Sciences, Department of Midwifery, Istanbul Topkapi University, Istanbul, Türkiye; 2 Department of Women's Health and Gynecologic Nursing, Florence Nightingale Faculty of Nursing, Istanbul University-Cerrahpasa, Istanbul, Türkiye

**Keywords:** clinical skills, knowledge retention, midwifery education, postpartum hemorrhage, randomized controlled trial, self-efficacy, simulation-based education

## Abstract

**Background:**

Postpartum hemorrhage is a time-sensitive obstetric emergency that requires rapid assessment and coordinated management. This study evaluated the effects of simulation-based education on midwifery students' postpartum hemorrhage management knowledge, skill performance, and self-efficacy**.**

**Methods:**

An assessor-blinded randomized controlled trial was conducted with 45 third-year midwifery students. Participants were randomly allocated to an intervention group that received traditional theoretical education followed by simulation-based education (*n* = 23) or a control group that received traditional theoretical education only (*n* = 22). Knowledge and self-efficacy were assessed before, immediately after, and four weeks after the intervention; skill performance was assessed immediately after and at four weeks.

**Results:**

Knowledge scores increased in both groups. A significant between-group difference was observed immediately after the intervention (*p* = 0.047), whereas the between-group difference at four weeks was not statistically significant (*p* = 0.541). In the linear mixed-effects analysis, the overall group × time interaction for knowledge was not significant [Wald *χ*^2^(2) = 2.56, *p* = 0.279]. Skill performance was substantially higher in the intervention group at both post-intervention assessments (both *p* < 0.001); the post-test mean difference was 8.83 points (95% CI 4.85–12.81; Cohen's *d* = 1.358), and the group × time interaction from post-test to four weeks was not significant (*p* = 0.742), indicating stability of the between-group difference over follow-up. Self-efficacy increased in both groups, with no significant overall group × time interaction [Wald *χ*^2^(2) = 3.10, *p* = 0.212].

**Conclusion:**

Simulation-based education was associated with a short-term between-group advantage in PPH-related knowledge and with a large, stable post-intervention advantage in skill performance across four weeks. No clear additional effect on self-efficacy was demonstrated. Because skill performance was not measured at baseline, the skill findings should be interpreted with appropriate caution.

**Clinical trial registration:**

ClinicalTrials.gov NCT07618442, https://clinicaltrials.gov/study/NCT07618442.

## 1 Introduction

Postpartum hemorrhage (PPH) remains a major cause of preventable maternal morbidity and mortality worldwide. Recent WHO guidance emphasizes the importance of accurate assessment and timely treatment bundles for PPH, reflecting its continued relevance as a global maternal health priority (1). PPH is commonly defined as cumulative blood loss of 1.000 mL or more, or blood loss accompanied by signs or symptoms of hypovolemia within 24 h after birth (2). Because PPH can progress rapidly, current international recommendations emphasize early recognition, standardized assessment, and coordinated clinical response (3, 4). These requirements make PPH management an important competency in nursing and midwifery education.

In nursing and midwifery education, preparing students for PPH management can be challenging because obstetric emergencies are complex, time-sensitive, and difficult to practice repeatedly in real clinical settings (5). Simulation-based education offers a structured way to address this gap by allowing learners to practice clinical assessment, decision-making, and emergency interventions in a controlled environment, with simulation design standards recommending that scenarios be developed according to learner needs, clear objectives, available resources, facilitation strategies, and planned evaluation methods (6). Recent PPH-related studies also suggest that simulation-based approaches may improve students' or clinicians' knowledge and performance, although outcomes vary according to study design, participant group, and educational context (7, 8).

Although recent studies generally report favorable effects of simulation-based education on knowledge and clinical performance in PPH management, findings related to self-efficacy, self-confidence, or perceived readiness remain variable. Recent PPH-focused studies have reported improvements in knowledge, skills, self-confidence, and satisfaction after simulation-based education; however, these outcomes have been examined using different participant groups, intervention formats, and follow-up periods (7–9). A recent systematic review of simulation-based learning in nursing education also identified long-term retention of knowledge and skills as an important methodological issue (10). Despite the growing evidence supporting simulation-based PPH education, uncertainty therefore remains regarding whether performance advantages persist over time and whether simulation produces comparable improvements in perceived self-efficacy.

This study aimed to evaluate the effects of simulation-based education on midwifery students' PPH management knowledge, skill performance, and self-efficacy.

## 2 Material and methods

### 2.1 Aim and hypotheses

The aim of this study was to determine the effects of simulation-based education on midwifery students' postpartum hemorrhage management knowledge, skill performance, and self-efficacy.

The study hypotheses were as follows:

H1^a^: The knowledge level of students in the intervention group will be higher than that of students receiving traditional theoretical education.
H1^b^: The skill performance level of students in the intervention group will be higher than that of students receiving traditional theoretical education.
H1^c^: The self-efficacy level of students in the intervention group will be higher than that of students receiving traditional theoretical education.

### 2.2 Study design

This study was conducted as an assessor-blinded randomized controlled trial to evaluate the effect of simulation-based education on midwifery students' knowledge, PPH management skill performance, and self-efficacy in postpartum hemorrhage management. Participants were randomly assigned to either the intervention group, which received traditional theoretical education followed by simulation-based education, or the control group, which received traditional theoretical education only. Knowledge and self-efficacy were assessed at three time points: before the intervention, immediately after the intervention, and four weeks after the intervention. PPH management skill performance was assessed immediately after the intervention and again at the four-week follow-up. The trial was registered at ClinicalTrials.gov (Registration No. NCT07618442).

The study was reported in accordance with the CONSORT 2025 statement for randomized trials (11). The participant flow throughout the trial is presented in Figure 1.

**Figure 1:**
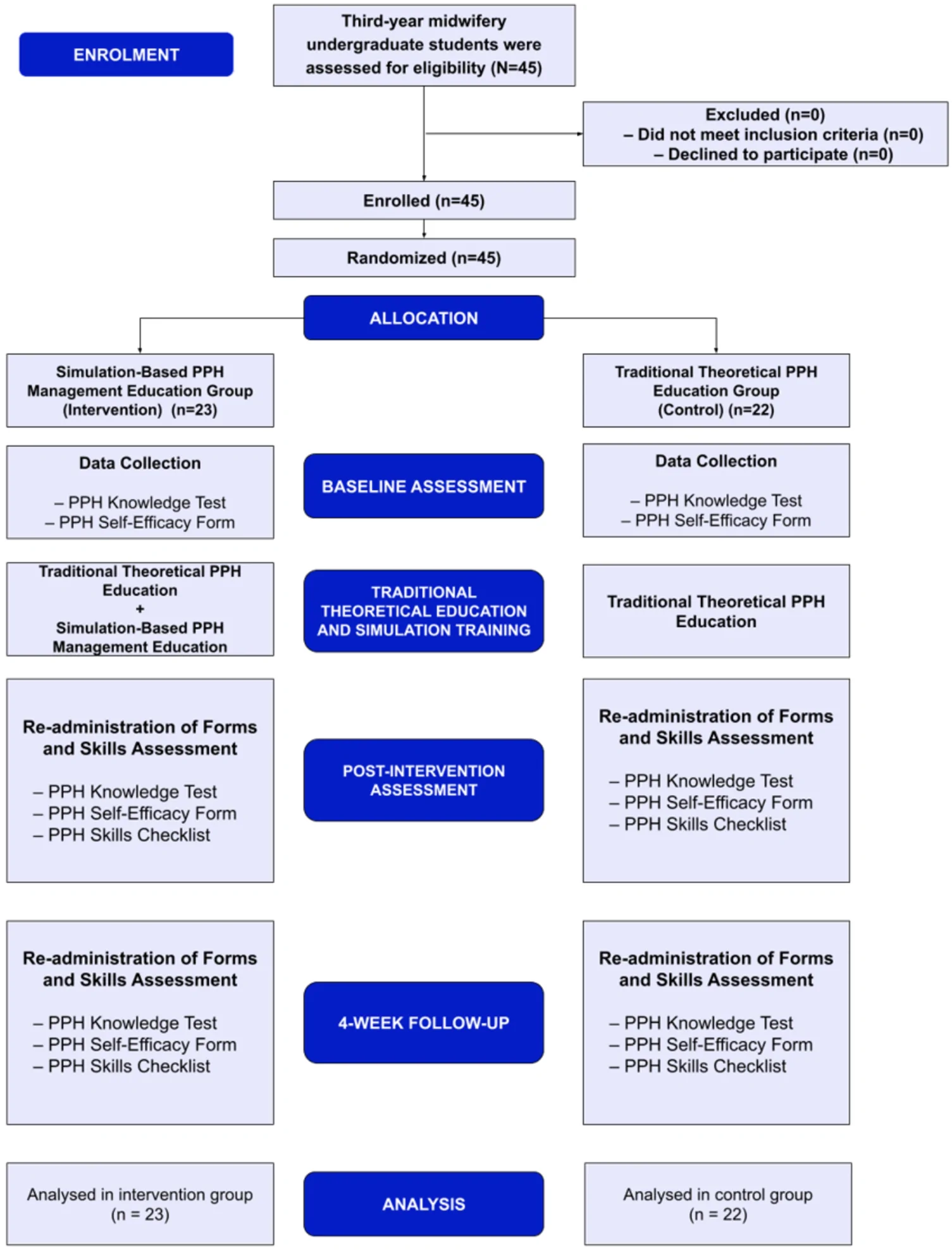
CONSORT flow diagram of participant enrollment, randomization, intervention, follow-up, and analysis.

### 2.3 Setting and participants

The study was conducted between May and June 2023 in the Nursing and Midwifery Simulation Laboratory of a university in Istanbul, Türkiye. The laboratory consisted of a simulation control room and a patient room separated by one-way glass. The patient room was equipped with a hospital bed, emergency cart, neonatal equipment, vital signs monitor, electronic fetal monitoring device, intravenous stands, and other materials required for postpartum hemorrhage management.

The study population consisted of third-year undergraduate midwifery students enrolled in the 2022–2023 academic year. Students were eligible to participate if they had completed the Normal Birth and Midwifery Care course and were currently enrolled in the High-Risk Birth and Midwifery Care course. Students who declined to participate or were not third-year midwifery students were excluded. An *a priori* sample-size calculation was performed in G*Power (version 3.1.9.7) using the between-group effect reported by Durmaz and Kakaşçı Gün (12) as the reference. The calculation was based on the Caring Assessment Questionnaire scores reported for the experimental and control groups (309.15 ± 17.58 vs. 255.42 ± 44.00), corresponding to an expected standardized effect size of Cohen's *d* = 1.607. A one-tailed independent-samples *t*-test (difference between two independent means) was specified with alpha = 0.05, power (1-beta) = 0.95, and a 1:1 allocation ratio. The resulting minimum sample size was 20 participants (10 per group; actual power approximately 0.964). Because this archived calculation was based on an independent-samples comparison, no repeated-measures correlation was specified, and no additional attrition adjustment was included.

A total of 45 eligible students participated in the study. All eligible students were included to reduce potential selection bias and to increase statistical power. No participants were excluded after enrollment or lost to follow-up. Participants were randomly assigned to the intervention group (*n* = 23) or the control group (*n* = 22).

### 2.4 Randomization and blinding

Randomization was conducted by entering anonymized student identification numbers into an online random sequence generator. Of the 45 students included in the study, 23 were allocated to the intervention group and 22 to the control group.

Because of the nature of the educational intervention, participants could not be blinded to whether they attended the simulation session. Therefore, the blinding strategy focused on outcome assessment. PPH management skill performance was assessed by two external observers who were blinded to group allocation and were not affiliated with the university where the participants were enrolled.

### 2.5 Data collection instruments

Data were collected using four instruments: a demographic information form, the Postpartum Hemorrhage Management Knowledge Test, the Postpartum Hemorrhage Management Self-Efficacy Form, and the Postpartum Hemorrhage Management Skills Checklist.

#### 2.5.1 Demographic information form

The demographic information form was developed by the researchers and included questions about participants' age and gender.

#### 2.5.2 Postpartum hemorrhage management knowledge test

The Postpartum Hemorrhage Management Knowledge Test was developed by the researchers based on national and international guidelines on postpartum hemorrhage management, including the American College of Obstetricians and Gynecologists Practice Bulletin on postpartum hemorrhage, the Royal College of Obstetricians and Gynaecologists Green-top Guideline No. 52, the World Health Organization recommendations for the prevention and treatment of postpartum hemorrhage, and the Turkish Ministry of Health Emergency Obstetric Care Management Guideline (2, 13–15) The ACOG bulletin discusses PPH risk factors, evaluation, prevention, and management; the RCOG guideline focuses on prevention and management of PPH; the WHO guideline provides general principles of PPH care; and the Turkish Ministry of Health guideline provides national emergency obstetric care guidance.

The initial version of the test consisted of 25 items and was reviewed by six experts. Following expert review and item analysis, two items with low discrimination indices were removed, resulting in a final 23-item test. Each correct answer was scored as 1 point, and incorrect answers were scored as 0. Total scores ranged from 0 to 23, with higher scores indicating higher knowledge of PPH management.

The test was piloted with 30 fourth-year midwifery students from another university. After the removal of two items, the Cronbach's alpha coefficient was 0.779. The content validity ratio and content validity index were 0.95 and 0.97, respectively.

#### 2.5.3 Postpartum hemorrhage management self-efficacy form

The Postpartum Hemorrhage Management Self-Efficacy Form was developed by the researchers to assess students' perceived self-efficacy in managing PPH. The form consisted of 13 items rated on a five-point Likert scale. Total scores ranged from 13 to 65, with higher scores indicating higher perceived self-efficacy.

The form was reviewed by six experts, and revisions were made according to their feedback. The content validity ratio and content validity index were 0.97 and 0.99, respectively.

#### 2.5.4 Postpartum hemorrhage management skills checklist

The Postpartum Hemorrhage Management Skills Checklist was developed by the researchers based primarily on the Turkish Ministry of Health Emergency Obstetric Care Management Guideline and supported by international PPH management recommendations (2, 13–15). The checklist consisted of 23 items assessing key steps in PPH management. Each item was scored as 0 = needs improvement, 1 = partially adequate, and 2 = adequate. Total scores ranged from 0 to 46, with higher scores indicating better PPH management skill performance.

The checklist was reviewed by six experts and revised according to their feedback. The content validity ratio and content validity index were both 1.00.

### 2.6 Intervention and simulation procedure

All participants first received traditional theoretical education on postpartum hemorrhage management. The education was delivered face-to-face by the researcher in a classroom setting and lasted approximately one hour. The content was based on the Postpartum Hemorrhage Management Information Booklet developed by the researchers in line with national and international guidelines. The session included the definition, importance, etiology, risk factors, prevention, treatment, and management of PPH. Theoretical education was supported by a PowerPoint presentation and included lecture, question–answer, discussion, and brainstorming methods.

Following theoretical education, participants were randomly allocated to the intervention and control groups. The intervention group received simulation-based PPH management education in addition to the theoretical session, whereas the control group received only the theoretical education.

The simulation-based intervention was developed and implemented in accordance with the International Nursing Association for Clinical Simulation and Learning Healthcare Simulation Standards of Best Practice™: Simulation Design (6). The learning objectives of the scenario were to enable students to assess vital signs, lochia, and pain in relation to PPH; implement emergency interventions and manage follow-up care; and communicate effectively during the emergency response. The scenario was conducted using a high-fidelity birthing simulator (SimMom, Laerdal Medical) in a simulation laboratory arranged as a postpartum patient room. The environment included a hospital bed, emergency cart, patient monitor, intravenous materials, neonatal equipment, and other materials required for PPH management.

Each simulation session consisted of a 5-minute prebriefing, a 15-minute simulation scenario, and a 30-minute debriefing session. During the scenario, a facilitator acted as a patient relative and provided cues when necessary to support the flow of the scenario without directly guiding students' clinical decisions. After the simulation, a structured debriefing session was conducted to consolidate learning and encourage reflection on students' performance.

The simulation was facilitated by a researcher who had completed formal training in simulation-based education.

Figure 2 presents an overview of the simulation environment, learning objectives, session flow, and realism-enhancing components of the postpartum hemorrhage scenario.

**Figure 2:**
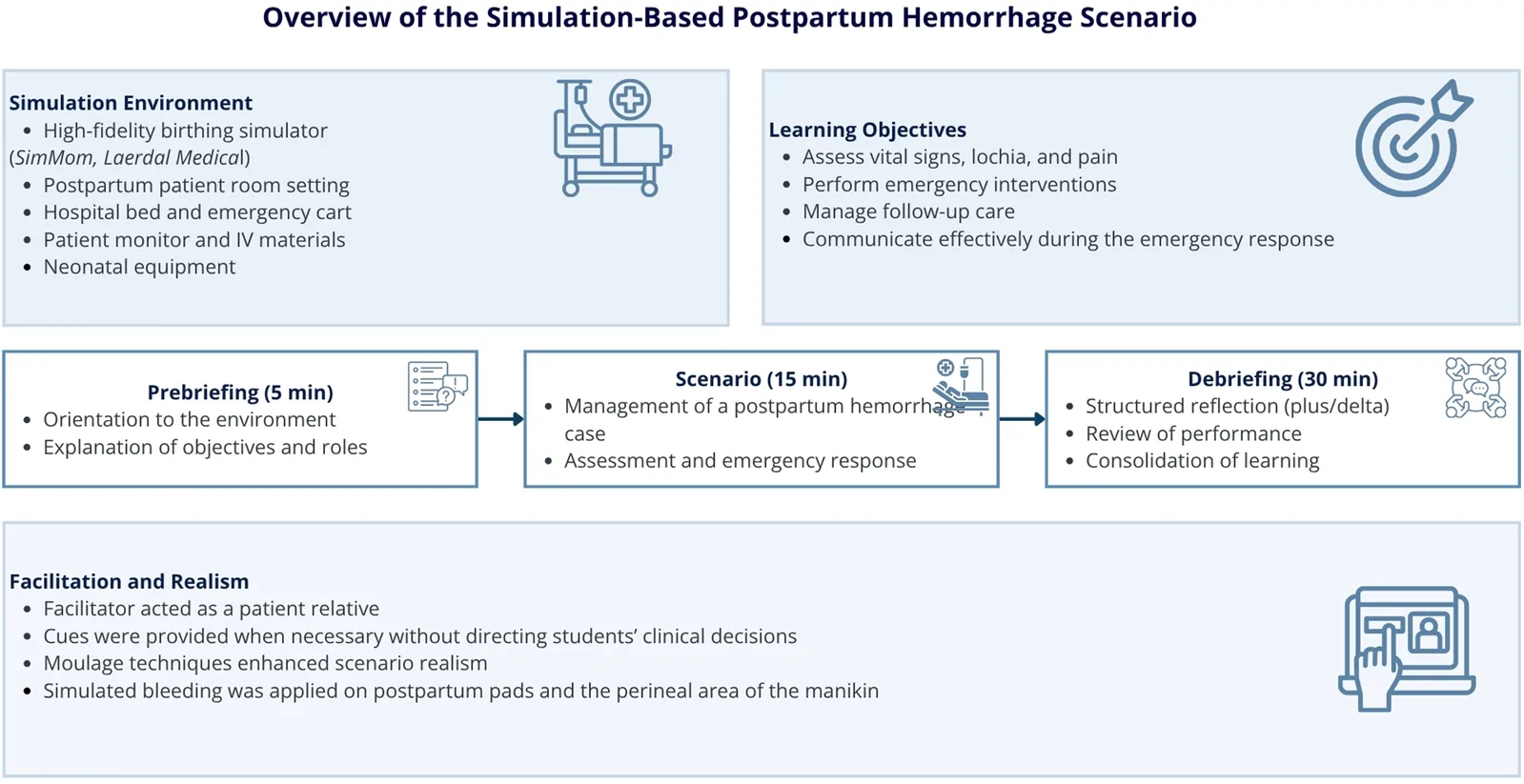
Overview of the simulation-based postpartum hemorrhage scenario.

The simulation scenario and educational materials were reviewed by six experts before implementation and revised according to their feedback. A pilot implementation was conducted before the main study to identify and correct practical issues related to the simulation environment, visibility of materials, accessibility of equipment, and scenario flow.

Moulage techniques were used to increase scenario realism, including simulated bleeding on postpartum pads and the perineal area of the manikin. The simulation environment and moulage setup, including simulated postpartum bleeding and facial sweating, are shown in Supplementary Figure S1.

### 2.7 Data collection procedure

Data were collected at three main time points: before the intervention, immediately after the intervention, and four weeks after the intervention. Before the educational intervention, all participants completed the demographic information form, the Postpartum Hemorrhage Management Knowledge Test, and the Postpartum Hemorrhage Management Self-Efficacy Form online. These measurements were considered baseline data. PPH management skill performance was not assessed at baseline; therefore, baseline equivalence between groups in skill performance could not be directly verified.

After baseline data collection, all participants received traditional theoretical education on PPH management. Following this session, participants were randomly allocated to the intervention and control groups. The intervention group then participated in simulation-based PPH management education, whereas the control group did not receive simulation-based training.

Immediately after the intervention period, both groups completed the Postpartum Hemorrhage Management Knowledge Test and the Postpartum Hemorrhage Management Self-Efficacy Form again. In the same week, both groups were invited individually for skill performance assessment using the Postpartum Hemorrhage Management Skills Checklist. Each student performed the required PPH management skills on a manikin, and the assessment lasted approximately 15 min per participant. Skill performance was assessed by two external observers with expertise in women's health and obstetric care. The observers were not affiliated with the university where the participants were enrolled, which was intended to reduce potential assessment bias related to student–instructor relationships.

Four weeks after the intervention, both groups completed the Postpartum Hemorrhage Management Knowledge Test and the Postpartum Hemorrhage Management Self-Efficacy Form for the third time. Skill performance was also reassessed using the same checklist, simulation environment, and observer procedure. This follow-up assessment was conducted to characterize short-term outcome patterns four weeks after the educational intervention.

To reduce the risk of contamination between groups, participants were invited to the simulation and assessment sessions by appointment. The intervention and control groups were scheduled separately, and efforts were made to prevent interaction between group members during the data collection process.

### 2.8 Statistical analysis

Original analyses were conducted using IBM SPSS Statistics version 27.0. Descriptive statistics, including frequency, percentage, mean, and standard deviation, were used to summarize participant characteristics and outcome scores. The normality of continuous variables was assessed using graphical methods, the Kolmogorov–Smirnov test, and the Shapiro–Wilk test.

For the original comparisons, independent-samples *t*-tests were used for normally distributed between-group comparisons and Mann–Whitney *U-*tests for variables that did not meet the normality assumption. Repeated-measures ANOVA was used for normally distributed within-group comparisons across measurement points, whereas the Friedman test and Wilcoxon signed-rank test were used for non-normally distributed repeated measurements. Chi-square tests were used for categorical variables. To directly evaluate whether outcome trajectories differed between groups, additional linear mixed-effects models were fitted to the participant-level data using Python 3.13 and statsmodels 0.14.6. Separate models were fitted for knowledge, self-efficacy, and skill performance, with a participant-specific random intercept and fixed effects for group, time, and the group × time interaction. Knowledge and self-efficacy models included baseline, immediate post-test, and four-week follow-up measurements. Because skill performance was not assessed at baseline, the skill model included the immediate post-test and four-week follow-up measurements only. Omnibus Wald *χ*^2^-tests were used to evaluate the group × time interaction.

The intraclass correlation coefficient (ICC) was calculated to evaluate inter-rater reliability for the Postpartum Hemorrhage Management Skills Checklist. The original reliability analysis used a two-way mixed-effects model with a consistency definition and average measures [ICC(3,k)], treating the two observers as fixed raters. The second-observer ratings were used for the inter-rater reliability analysis; the primary skill-performance analyses were based on the checklist total score recorded in the main study dataset and were not computed as an average of the two observers' scores. Effect sizes were calculated using Cohen's d to estimate the magnitude of between-group differences. Statistical significance was set at *p* < 0.05.

### 2.9 Ethical considerations

Ethical approval for the study was obtained from the Non-Invasive Clinical Research Ethics Committee of Istanbul Atlas University (Approval No: E-22686390-050.99-24891). Institutional permission was also obtained for the use of the simulation laboratory.

All participants were informed about the aim, procedures, voluntary nature of participation, and confidentiality of the study. Written informed consent was obtained from all participants before data collection. Participants were informed that they could withdraw from the study at any time without any academic penalty. Personal data were anonymized, and all data were stored securely. The study was conducted in accordance with the principles of the Declaration of Helsinki.

## 3 Results

### 3.1 Participant characteristics

A total of 45 third-year midwifery students participated in the study, with 23 students allocated to the intervention group and 22 to the control group. All participants were female (Table 1).

**Table 1:** Participant characteristics.

Variable	Intervention group (*n* = 23)	Control group (*n* = 22)	Total (*N* = 45)	Test statistic	*P*
Age, M ± SD	21.58 ± 0.86	21.52 ± 0.89	21.55 ± 0.87	*t* = 0.399ᵃ	0.690
Female, *n* (%)	23 (100.0)	22 (100.0)	45 (100.0)	—	—

ᵃ Independent samples *t*-test.

### 3.2 Inter-rater reliability of the skills checklist

Inter-rater reliability between the two external observers was assessed using a two-way mixed-effects, consistency, average-measures ICC [ICC(3,k)]. The ICC was 0.712 (95% CI: 0.321–0.878) for Phase I and 0.785 (95% CI: 0.492–0.909) for Phase II (Table 2), indicating acceptable-to-good consistency of the average ratings. Mean scores differed between raters (Phase I: 15.08 vs. 18.08; Phase II: 10.47 vs. 12.26), so the ICC estimates should be interpreted as measures of consistency rather than absolute agreement.

**Table 2:** Inter-rater reliability of the postpartum hemorrhage management skills checklist {two-way mixed-effects, consistency, average-measures ICC [ICC(3,k)]}.

Phase	Rater 1, M ± SD	Rater 2, M ± SD	ICC	95% CI
Phase I	15.08 ± 5.15	18.08 ± 3.83	0.712	0.321–0.878
Phase II	10.47 ± 4.23	12.26 ± 3.86	0.785	0.492–0.909

### 3.3 Knowledge scores

Knowledge scores increased over time in both groups (Table 3). The groups did not differ significantly at baseline (*p* = 0.357). The intervention group had a higher mean knowledge score immediately after the intervention (13.39 ± 3.79 vs. 11.00 ± 4.07; *p* = 0.047), whereas the between-group difference at four weeks was not statistically significant (12.13 ± 2.49 vs. 11.50 ± 4.19; *p* = 0.541). In the linear mixed-effects model, the overall group × time interaction was not statistically significant [Wald *χ*^2^(2) = 2.56, *p* = 0.279]. Relative to baseline, the additional post-test change in the intervention group was 1.57 points (95% CI −0.80 to 3.94; *p* = 0.194), and the additional four-week change was −0.19 points (95% CI −2.56 to 2.18; *p* = 0.875). Thus, the time-point comparison at immediate post-test should be interpreted as a localized between-group difference rather than evidence of a statistically confirmed differential trajectory across all three measurement points.

**Table 3:** Knowledge scores and between-group comparisons by measurement time.

Group/Comparison	Pre-test M ± SD	Post-test M ± SD	Four-week follow-up M ± SD	Within-group test	Within-group *p*
Intervention	9.96 ± 3.20	13.39 ± 3.79	12.13 ± 2.49	F = 8.587ᵃ	**<0.001** ***
Control	9.14 ± 2.68	11.00 ± 4.07	11.50 ± 4.19	F = 4.084ᵃ	**0.023** *
Between-group test	*t* = 0.93ᵇ	*t* = 2.04ᵇ	*t* = 0.62ᵇ	—	—
Between-group p	0.357	**0.047** *	0.541	—	—
Cohen's d	0.278	0.609	0.184	—	—
Group × time interaction (mixed model)	—	—	—	Wald *χ*²(2) = 2.56	0.279

Bold values indicate statistically significant *p*-values (*p* < 0.05).

ᵃ Repeated measures ANOVA.

ᵇ Independent samples *t*-test.

* *p* < 0.05.

*** *p* < 0.001.

### 3.4 Postpartum hemorrhage management skill performance

PPH management skill performance scores were significantly higher in the intervention group than in the control group both immediately after the intervention and at the four-week follow-up (Table 4). At post-test, the between-group mean difference was 8.83 points (95% CI 4.85–12.81; *p* < 0.001), corresponding to a large effect size (Cohen's *d* = 1.358). The between-group difference also remained statistically significant at four weeks (*p* < 0.001; Cohen's *d* = 1.407). In the linear mixed-effects model, the estimated between-group difference at post-test was 8.83 points (95% CI 5.26–12.40; *p* < 0.001), and the group × time interaction from post-test to four weeks was not significant [*β* = −0.82, 95% CI −5.67 to 4.03; Wald *χ*^2^(1) = 0.11, *p* = 0.742], indicating that the magnitude of the between-group difference did not change significantly during follow-up. Because skill performance was not assessed at baseline, these post-intervention differences cannot establish baseline skill equivalence and should be interpreted together with the randomized design and this measurement limitation.

**Table 4:** PPH management skill performance scores by group and measurement time.

Group/Comparison	Post-test M ± SD	Four-week Follow-up M ± SD	**Within-group p**
Intervention	37.73 ± 4.91	37.69 ± 5.01	0.909^a^
Control	28.90 ± 7.83	29.68 ± 6.33	0.673^a^
Between-group test	*t* = 4.509ᵇ	U = 420.0ᵉ	—
Between-group *p*	**<0.001** ***	**<0.001** ***	—
Mean difference (95% CI)	8.83 (95% CI 4.85–12.81)	—	—
Cohen's d	1.358	1.407	—
Group × time interaction (mixed model)	—	Wald χ²(1) = 0.11	0.742

Bold values indicate statistically significant *p*-values (*p* < 0.05).

a Wilcoxon signed-rank test.

ᵇ Independent samples *t*-test.

ᵉ Mann–Whitney *U*-test.

*** *p* < 0.001.

### 3.5 Self-efficacy scores

Self-efficacy scores increased over time in both groups (Table 5). The intervention group increased from 45.73 ± 8.62 at baseline to 51.65 ± 9.90 immediately after the intervention and 52.34 ± 12.29 at four weeks. The control group increased from 43.95 ± 8.36 to 46.18 ± 10.64 and then to 51.95 ± 10.86. Although the between-group difference was larger immediately after the intervention (Cohen's *d* = 0.533; *p* = 0.081), the groups were very similar at four weeks (Cohen's *d* = 0.034; *p* = 0.910). The linear mixed-effects model showed no significant overall group  ×  time interaction [Wald *χ*^2^(2) = 3.10, *p* = 0.212]. The additional post-test change associated with the intervention group was 3.69 points (95% CI −2.15 to 9.53; *p* = 0.216), while the additional four-week change relative to baseline was −1.39 points (95% CI −7.23 to 4.45; *p* = 0.640).

**Table 5:** Self-efficacy scores and between-group comparisons by measurement time.

Group/comparison	Pre-test M ± SD	Post-test M ± SD	Four-week follow-up M ± SD	Within-group test	Within-group *p*
Intervention	45.73 ± 8.62	51.65 ± 9.90	52.34 ± 12.29	χ^2^ = 21.86^a^	**<0.001** ***
Control	43.95 ± 8.36	46.18 ± 10.64	51.95 ± 10.86	χ² = 7.61^a^	**0.022** *
Between-group test	*t* = 0.70ᵇ	*t* = 1.79ᵇ	*t* = 0.11ᵇ	–	–
Between-group *p*	0.485	0.081	0.910	–	–
Cohen's d	0.210	0.533	0.034	–	–
Group × time interaction (mixed model)	–	–	–	Wald χ^2^(2) = 3.10	0.212

Bold values indicate statistically significant *p*-values (*p* < 0.05).

a Friedman test.

ᵇ Independent samples *t*-test.

* *p* < 0.05.

*** *p* < 0.001.

## 4 Discussion

This randomized controlled trial evaluated the effect of simulation-based education on midwifery students' PPH management knowledge, skill performance, and self-efficacy. Knowledge scores increased over time in both groups, and a significant between-group difference was observed immediately after the intervention; however, the mixed-effects analysis did not identify a significant overall group × time interaction for knowledge. The clearest finding was the large difference in PPH management skill performance, with substantially higher scores in the intervention group at both post-intervention assessments. The nonsignificant skill group × time interaction indicated that this between-group difference remained statistically stable over the four-week follow-up. Self-efficacy increased in both groups, but neither the time-point comparisons nor the mixed-effects model demonstrated a significant additional longitudinal effect of simulation.

The higher immediate post-test knowledge score in the intervention group is consistent with previous studies reporting that simulation-based education can improve PPH-related knowledge among students and clinicians. Alsaraireh et al. (16) found that a high-fidelity simulation-based teaching-learning experience improved maternal health nursing students' knowledge of PPH and was associated with favorable confidence and satisfaction outcomes. Similarly, Faustine et al. (8) reported improvements in knowledge, skills, self-confidence, and satisfaction among student nurse-midwives after simulation-based PPH education. Among practicing nurses, Abd Elhakm and Elbana (17) and Almutairi et al. (7) also reported improvements in knowledge following simulation-based PPH training. In the present study, the between-group difference observed immediately after training was not statistically significant at the four-week assessment, and the overall group × time interaction was also not significant. Accordingly, the results support an immediate between-group difference but do not establish a statistically distinct longitudinal knowledge trajectory attributable to the intervention. The moderate absolute post-test knowledge score in the intervention group should also be interpreted carefully. PPH management requires integration of bleeding recognition, clinical assessment, pharmacological management, emergency response, communication, and documentation; therefore, the findings indicate short-term improvement rather than complete cognitive mastery.

The most prominent finding of this study was the large between-group difference in PPH management skill performance. The intervention group had substantially higher skill scores than the control group at both post-test and four-week follow-up, with large effect sizes. The post-test mean difference was 8.83 points (95% CI 4.85–12.81), and the mixed-effects model estimated a similar post-test group difference of 8.83 points (95% CI 5.26–12.40). Importantly, the group × time interaction from post-test to four weeks was not significant, indicating that the magnitude of the between-group difference remained stable during follow-up rather than converging or diverging significantly. This pattern is consistent with previous research suggesting that simulation is particularly useful for procedural and performance-based learning in obstetric emergencies (9, 17–19). Aker and Aydın Kartal's randomized parallel-group trial is especially relevant because it examined high-fidelity, scenario-based PPH simulation among third-year midwifery students. The use of moulage, simulated bleeding, emergency equipment, monitoring devices, and clinical documentation forms may have increased scenario realism and supported the translation of theoretical knowledge into observable performance. Nevertheless, because skill performance was not measured before the intervention, the present study cannot directly verify baseline equivalence in PPH skill. The post-intervention skill differences are therefore strongly suggestive of an educational advantage but should be interpreted together with this baseline measurement limitation.

Self-efficacy findings were more nuanced. Self-efficacy increased in both groups, and the intervention group showed a larger numerical advantage immediately after training; however, the control group approached the intervention group by four weeks. Neither the time-point comparisons nor the mixed-effects group × time analysis showed a statistically significant additional longitudinal effect of simulation on self-efficacy. Previous studies have reported variable confidence-related outcomes. Alsaraireh et al. (16), Faustine et al. (8), and Abd Elhakm and Elbana (17) reported favorable confidence or self-confidence outcomes after simulation-based PPH education. One possible explanation for the present pattern is that repeated completion of the questionnaires and repeated manikin-based assessments may have influenced participants' perceived competence in both groups. It is also possible that realistic simulation increases awareness of one's limitations while simultaneously supporting confidence development. These explanations are speculative and should be investigated in studies specifically designed to examine mechanisms underlying self-efficacy change.

Several methodological features strengthen this study. The randomized controlled design, planned sample-size calculation, four-week follow-up, and use of two external observers support the rigor of the study. The observers were not affiliated with the students' university, which may have reduced assessment bias related to student–instructor relationships. Inter-rater reliability was also assessed, increasing transparency regarding skill performance scoring. In addition, the simulation was developed according to healthcare simulation standards and facilitated by a researcher who had completed formal simulation training.

This study also has limitations. It was conducted with a relatively small sample from a single university, which may limit generalizability and reduce power for detecting interaction effects. Baseline characteristics were limited to age and gender; previous exposure to obstetric emergencies, academic achievement, and prior simulation experience were not assessed. Importantly, skill performance was not measured before the intervention, so baseline equivalence in PPH management skill could not be verified despite random allocation. In addition, the same knowledge and self-efficacy instruments were administered repeatedly, creating the possibility of testing or practice effects that may have contributed to improvements in either group. Although participant-level linear mixed-effects models were added to directly test group × time interactions, the modest sample size means that nonsignificant interaction tests should still be interpreted with attention to uncertainty and confidence intervals. Although the groups were scheduled separately, participants were from the same academic year, and informal communication during the four-week follow-up period could not be fully prevented. Inter-rater reliability was evaluated using a consistency rather than an absolute-agreement ICC, and the two raters showed some systematic differences in mean scores; therefore, the reliability estimates should not be interpreted as evidence of identical absolute scoring. Skill performance was assessed in a simulated environment rather than in clinical practice, and self-efficacy was measured using a researcher-developed form. Future studies should include multi-center designs, larger samples, baseline assessment of clinical skill, and validated self-efficacy instruments.

## 5 Conclusion

Simulation-based education was associated with a significant between-group advantage in PPH-related knowledge immediately after training; however, the overall knowledge group × time interaction was not statistically significant, and the between-group knowledge difference was not significant at four weeks. The clearest finding was the large difference in PPH management skill performance, which favored the intervention group at both post-intervention assessments and remained stable over the four-week follow-up in the mixed-effects analysis. Self-efficacy increased in both groups, but no significant additional longitudinal effect of simulation was demonstrated.

The large and stable post-intervention differences in skill performance support simulation as a complementary strategy for emergency skill training in undergraduate midwifery education. However, because skill performance was not assessed at baseline, the study cannot directly establish pre-intervention equivalence in skill, and causal interpretation of the post-intervention skill difference should remain appropriately cautious.

Future research should determine whether repeated or longitudinal simulation exposure improves the durability of knowledge gains or produces additional benefits for self-efficacy, using larger multi-center samples, baseline skill assessment, validated outcome measures, and adequately powered longitudinal analyses.

## Data Availability

The raw data supporting the conclusions of this article will be made available by the authors, without undue reservation.
