# Supplementary material for: Simulation-based education improves postpartum hemorrhage management knowledge and skills: a randomized controlled trial
Source: Front Med (Lausanne). 2026 Sep 29;13:1939595. doi: 10.3389/fmed.2026.1939595 (PMC13657912; doi:10.3389/fmed.2026.1939595)
Supplement: Supplementary file 1 [file Table1.docx]

Supplementary Material

## 1.1 Supplementary Figures

**Supplementary Figure 1. Simulation environment and moulage setup used in the postpartum hemorrhage scenario.**

**Alt Text:** Four-panel photograph showing a high-fidelity birthing simulator in a hospital bed during a postpartum hemorrhage scenario. Simulated blood is visible on the perineal area and absorbent pads, and facial sweating is represented to enhance clinical realism.
